# *KRAS* testing of patients with metastatic colorectal cancer in a community-based oncology setting: a retrospective database analysis

**DOI:** 10.1186/s13046-015-0146-5

**Published:** 2015-03-27

**Authors:** Gebra Cuyun Carter, Pamela B Landsman-Blumberg, Barbara H Johnson, Paul Juneau, Steven J Nicol, Li Li, Veena Shankaran

**Affiliations:** Eli Lilly and Company, Lilly Corporate Center, Indianapolis, IN 46285 USA; Truven Health Analytics, Bethesda, MD USA; University of Washington, Seattle, WA USA

**Keywords:** KRAS, KRAS testing, Metastatic colorectal cancer, mCRC

## Abstract

**Background:**

In 2009, treatment guidelines were updated to recommend *KRAS* testing at diagnosis for patients with metastatic colorectal cancer (mCRC). We investigated *KRAS* testing rates over time and compared characteristics of *KRAS*-tested and not-tested patients in a community-based oncology setting.

**Methods:**

Adult patients with a diagnosis of mCRC from 2008–2011 were selected from the ACORN Data Warehouse (ACORN Research LLC, Memphis, TN). Text mining of physician progress notes and full chart reviews identified *KRAS*-tested patients, test dates, and test results (*KRAS* status). The overall proportion of eligible patients *KRAS*-tested in each calendar year was calculated. Among *KRAS*-tested patients, the proportion tested at diagnosis (within 60 days) was calculated by year. Univariate and multivariate analyses were used to compare patient characteristics at diagnosis between tested and not-tested cohorts, and to identify factors associated with *KRAS* testing.

**Results:**

Among 1,363 mCRC patients seen from 2008–2011, 648 (47.5%) were *KRAS*-tested. Among newly diagnosed mCRC patients, the rate of *KRAS* testing increased from 5.9% prior to 2008, to 13.9% in 2008, and then jumped dramatically to 32.3% in 2009, after which a modest yearly increase continued. The proportions of *KRAS*-tested patients who had been diagnosed in previous years but not tested previously increased from 17.7% in 2008 to 27.0% in 2009, then decreased to 19.0% in 2010 and 17.6% in 2011. Among patients who were *KRAS*-tested, the proportions tested at the time of diagnosis increased annually (to 78.4% in 2011). Patients more likely to have been tested included those with lung metastases, poor performance status, more comorbidities, and mCRC diagnosis in 2009 or later.

**Conclusions:**

The frequency of *KRAS* testing increased over time, corresponding to changes in treatment guidelines and epidermal growth factor receptor inhibitor product labels; however, approximately 50% of eligible patients were untested during the study period.

## Background

Colorectal cancer (CRC) is the third leading cause of cancer deaths in the United States (US), with an estimated 142,820 new cases to be diagnosed in 2013 [1]. At the time of diagnosis, approximately 39% of patients with CRC present with localized disease (stages I-II), 37% present with regional metastases (stage III), and 19% with distant metastases (stage IV, or metastatic CRC [mCRC]) [2]. In mCRC, common sites for metastases include the liver, peritoneum, and lung. The 5-year survival rate for patients diagnosed with mCRC is approximately 10% to 12% [1,3].

Conventional chemotherapies used to treat mCRC include combinations of 5-fluorouracil, leucovorin, capecitabine, oxaliplatin, and irinotecan [4]. Bevacizumab (Avastin) is also indicated for treatment of mCRC in combination with conventional chemotherapies [5]. In addition, targeted therapies that interfere with epidermal growth factor receptor (EGFR)-mediated pathways (i.e., EGFR inhibitors) have been developed for the treatment of mCRC. In 2004, the EGFR inhibitor cetuximab (Erbitux) was approved in the US for the second-line treatment of mCRC in patients whose tumors overexpress EGFR [6], and in 2006, the EGFR inhibitor panitumumab (Vectibix) was approved for the second-line treatment of mCRC in patients with disease progression on or following conventional chemotherapies [7]. Since then, cetuximab has had its indication revised to include first- and second-line treatment of mCRC, in combination with conventional chemotherapies or as a single agent in patients who have failed oxaliplatin- and irinotecan-based therapy [6]. Similarly, in 2014, panitumumab had its indication revised to include first-line treatment of mCRC, in combination with conventional chemotherapies or as a single agent in patients who have failed fluoropyrimidine therapy [7].

*KRAS* is an oncogene involved with the EGFR signaling pathway; *KRAS* activity in mCRC is associated with increased cell proliferation, angiogenesis, migration, and survival of the cancer tissue [4,8]. In mCRC, only tumors carrying the normal (or wild-type [WT]) allele of the *KRAS* oncogene (i.e., *KRAS* mutation-negative) may have favorable responses to these EGFR inhibitors, and patients whose tumors carry certain *KRAS* mutations do not derive any benefit [8-10]. Approximately 60% of patients with mCRC carry the WT *KRAS* allele, and thus these patients may be appropriate candidates for therapy with EGFR inhibitors [4,8,9]. A recent study has suggested that *KRAS* mutation status is also predictive of long-term prognosis among patients with mCRC treated by conventional chemotherapy [11].

In light of these findings, the National Comprehensive Cancer Network (NCCN) updated its treatment guidelines for mCRC in late 2008, recommending that *KRAS* testing on tumors and metastases be part of the pretreatment workup for all patients with metastatic (stage IV) disease [4,12,13]. In mid-2009, NCCN updated these guidelines, stipulating that only patients with WT *KRAS* genotypes should receive treatment with EGFR inhibitors [13-15]. Similarly, the American Society for Clinical Oncology (ASCO) recommended that all patients with mCRC who are candidates for anti-EGFR antibody therapy have their tumors tested for *KRAS* mutations, and that those in whom mutations in codon 12 or 13 are detected should not receive EGFR inhibitors [15,16]. In 2009, the US Food and Drug Administration (FDA) required that the labels for both cetuximab and panitumumab be updated to restrict these agents to patients with mCRC whose tumors carry the WT KRAS genotype [6,7,17]. Most recently, in 2012, the FDA approved the first companion diagnostic test for *KRAS* genotyping (the therascreen® RGQ PCR Kit).

The objectives of the present study were to investigate *KRAS* testing rates over time and to compare the characteristics of tested and not-tested patients in a sample of community-based US oncology practices. In recent years, the clinical uptake and utilization of *KRAS* testing in mCRC, and the impact of *KRAS* testing on treatment outcomes, have been investigated in observational studies performed in Europe, Latin America, Asia [18], and the US [19]. In contrast to previous US studies, the current analysis includes patients covered by multiple healthcare payers (e.g., commercial, Medicare, Medicaid) enrolled in various plan types as well as self-pay patients.

## Methods

### Data source

The primary data source for this study was the ACORN EMR Warehouse (ACORN Research LLC, Memphis, TN), a database which contains information on approximately 175,000 patients seen in 12 community-based oncology practices by 120 oncologists across the US since 2004. This database links electronic medical records (EMRs), which contain patient demographics, tumor type and stage, treatments, and outcomes, with laboratory results and other clinical information. One limitation is that the results of *KRAS* testing are not captured in standard EMR data fields; however, this information is often found in physician progress notes, which ACORN stores electronically in the data warehouse to complement the EMR data. Deidentified data on patients meeting select criteria were provided by ACORN for analysis.

### Study population

Adult patients (aged 18 years or older) with a primary diagnosis of mCRC (based on ICD-9-CM codes [CRC: ICD-9-CM 153.x-154.1; metastatic disease: ICD-9-CM 197.x-198.x] and/or listing of “stage IV” or “stage 4” in physician notes) between January 1, 2008 and December 31, 2011 were identified. Patients with evidence of other primary cancers and those with only one visit to a contributing practice were excluded. All database records for included patients were extracted through March 31, 2012. For the purposes of the present study, “baseline” was defined as the time of first diagnosis of metastatic disease.

### Development and validity of *KRAS* testing algorithms

Algorithms for electronic text mining of physician progress notes were developed by ACORN to identify whether patients with mCRC were tested for *KRAS* and whether a patient’s tumor designation was WT or mutant [20]. These text mining algorithms were validated by trained oncology nurse abstractors who reviewed a random selection of 300 charts. When applied to the dataset, the best-performing algorithm (employing a random forest approach) achieved a kappa value (i.e., how often the model agreed with the chart review, accounting for chance) of 0.97, with a positive predictive value of 96.4% and a negative predictive value of 98.0% [20]. Trained oncology nurses then reviewed the medical charts of all patients identified by the model as “*KRAS*-tested” and recorded the results (WT, mutant, or unknown [ie, no evidence of testing results]) and the date of testing. The data were then merged with the study population data using blinded patient identifiers.

### Proportion of population tested

The overall rate of *KRAS* testing was based on the number of patients tested at any point between diagnosis of CRC and end of follow-up, divided by the total study sample. Rate of *KRAS* testing by year was based on the number of patients tested in the given year divided by the number of patients eligible for the given year. Patients eligible for testing, by year, was the sum of patients newly diagnosed with mCRC within a given year and those diagnosed with mCRC in previous years (with a visit to a participating provider within the given year) but not previously *KRAS*-tested. Finally, the proportion of those tested at diagnosis was defined as those tested within 60 days (before or after) of their first recorded mCRC diagnosis date within a given year divided by the total number of study patients diagnosed with mCRC in that same year.

### Statistical analyses

Demographic information, disease characteristics, and clinical history at first diagnosis of metastatic disease were compared between *KRAS*-tested and not-tested patients using chi-squared and Student’s t-tests. A logistic regression model was used to identify baseline factors associated with ever being *KRAS*-tested and with being tested at diagnosis. The level of statistical significance for all tests was 0.05 and results were not adjusted for multiple comparisons. All statistical analyses were performed using SAS version 9.2, on a PC platform.

## Results

Among 12,420 patients with a CRC diagnosis between 2008 and 2011, 2,080 were 18 years of age or older with documented distant metastases. Of these patients, 686 were excluded because they had evidence of other cancers, and an additional 31 were excluded because they only had one visit to the contributing practice, yielding a study population of 1,363 patients (Table 1). Of these 1,363 patients, 648 (47.5%) were *KRAS*-tested at some point in their disease. Of these 648 tested patients, 312 patients (48.1%) were identified as WT, 274 patients (42.3%) as mutant, and 62 patients (9.6%) as unknown (no evidence of testing results). Proportions of those patients *KRAS*-tested were similar between males and females, but *KRAS* testing was more frequent among patients aged 65 years and older than among younger patients (*p* = 0.002). Patients tested for *KRAS* were also less likely to be self-pay (3.9% vs. 8.1% for not-tested patients, *p* = 0.040), were more likely to be enrolled in a clinical trial (13.7% vs. 8.7%, *p* = 0.003), had more comorbid conditions (mean, 3.7 vs. 2.7, *p* < 0.001), and had poorer performance status (ECOG 2–4: 41.9% vs. 34.0%, *p* = 0.042) at diagnosis. No significant differences were found between tested and nontested cohorts for race, gender, cancer location (colon, rectum, or both), tumor histologic grade, or whether patients were newly diagnosed versus having recurrent mCRC.

**Table 1:** **Demographic characteristics of the eligible study population**

**Parameter**	**Number of patients (** ***N*** **= 1,363)**
**Age, years**	
18-34	26 (1.9%)
35-54	357 (26.2%)
55-74	724 (53.1%)
≥75	256 (18.8%)
**Gender**	
Female	640 (47.0%)
Male	723 (53.0%)
**Race**	
Caucasian	724 (53.1%)
African American	182 (13.4%)
Hispanic	8 (0.6%)
Other	23 (1.7%)
Not recorded	426 (31.3%)
**Insurance type**	
Commercial	663 (48.6%)
Medicare	461 (33.8%)
Medicaid	105 (7.7%)
Self-pay	83 (6.1%)
Other	51 (3.7%)
**Death reported**	653 (47.9%)
**Enrolled in a clinical trial**	151 (11.1%)
**Location of cancer**	
Colon	915 (67.1%)
Rectum	397 (29.1%)
Both	51 (3.7%)
**Course of mCRC**	
Newly diagnosed	482 (35.4%)
Recurrent	43 (3.2%)
Other	4 (0.3%)
Not reported	834 (61.2%)
**Consolidated/Combined ECOG/KPS score**	
ECOG = 0 to 1/KPS = 80 to 100	466 (34.2%)
ECOG = 2-4/KPS = 10-70	274 (20.1%)
Not reported	623 (45.7%)
**Histologic grade**	
Well differentiated	40 (2.9%)
Moderately differentiated	265 (19.4%)
High grade/poorly differentiated	89 (6.5%)
Undifferentiated	3 (0.2%)
Cannot be assessed	30 (2.2%)
Not reported	936 (68.7%)
***KRAS*** **genotype**	
Wild-type	312 (22.9%)
Mutant	274 (20.1%)
Unknown	62 (4.5%)
Not tested	715 (52.5%)

*Abbreviations*: *ECOG* Eastern Cooperative Oncology Group, *KPS* Karnofsky performance score, *mCRC* metastatic colorectal cancer.

**Figure 1:**
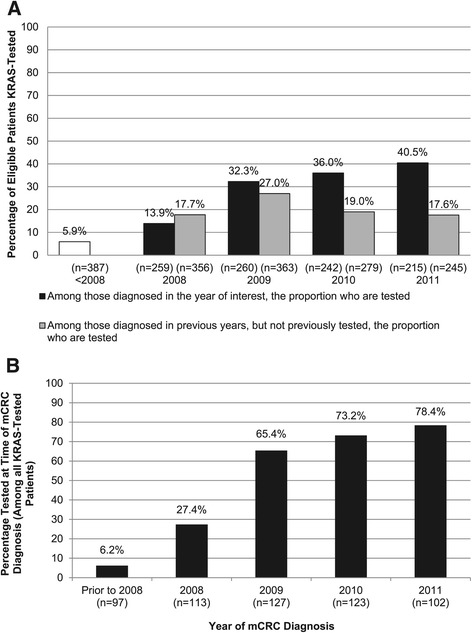
**KRAS testing by calendar year.** Proportion of newly diagnosed patients with mCRC tested for *KRAS* genotype and proportion of patients diagnosed in a prior year but not previously tested for *KRAS* genotype by calendar year **(A)**, and among *KRAS*-tested patients, proportion tested at time of mCRC diagnosis by calendar year **(B)**. Abbreviation: mCRC = metastatic colorectal cancer.

The proportions of eligible patients ever *KRAS*-tested increased from 5.9% to 16.1% in the calendar year 2008 (prior to the inclusion of testing recommendations in the NCCN guidelines) and continued to increase to 29.1% in 2009 (coincident with NCCN and ASCO guidance updates and US labeling changes regarding *KRAS* genotype for cetuximab and panitumumab), after which the proportions of tested patients remained fairly consistent. However, as shown in Figure 1A, when looking separately at the two populations of eligible patients, the proportions of newly diagnosed mCRC patients *KRAS*-tested increased from 13.9% in 2008 to 40.5% in 2011, while patients who were previously diagnosed, but not tested, tended to remain not tested over time. Among patients who were *KRAS*-tested, the proportions of those tested at the time of diagnosis increased greatly over the four years testing was widely available (27.4% in 2008 to 78.4% in 2011) (Figure 1B).

**Figure 2:**
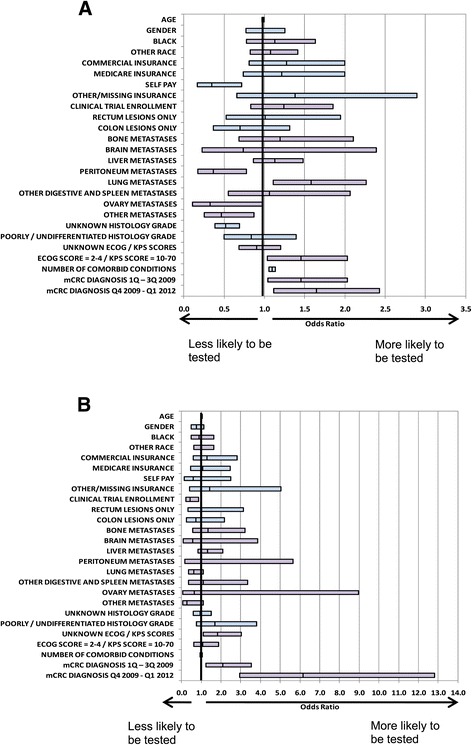
**Baseline characteristics associated with** ***KRAS*** **testing.** From multivariate logistic regression modeling among all eligible patients **(A)** and among *KRAS*-tested patients, those tested at time of mCRC diagnosis **(B)**. Reference groups: gender-male, race-Caucasian, insurance-Medicaid, location of lesions-both, histology grade-high/moderately differentiated, ECOG/KPS score-(0-1/80-100), mCRC diagnosis-2008 or earlier. Abbreviations: ECOG = Eastern Cooperative Oncology Group; KPS = Karnofsky performance score; mCRC = metastatic colorectal cancer; Q = quarter.

Baseline characteristics believed to be associated with ever being *KRAS*-tested, and with being *KRAS*-tested at diagnosis (among those tested), were modeled in a multivariable model (Figure 2A and B, respectively). Among eligible patients, those more likely to have been *KRAS*-tested (Figure 2A) included those with lung metastases (OR: 1.58, 95% CI: 1.11 to 2.26; ref: those with no lung metastases), those with poor performance status (OR: 1.45, 95% CI: 1.04 to 2.03; ref: those with good performance status), those with more comorbid conditions (OR: 1.10, 95% CI: 1.06 to 1.14), those diagnosed during the first three quarters of 2009 (OR: 1.46, 95% CI: 1.04 to 2.03; ref: those diagnosed in 2008 or earlier), and those diagnosed in the fourth quarter of 2009 or later (OR: 1.65, 95% CI: 1.12 to 2.43; ref: those diagnosed prior to the fourth quarter of 2009). Those less likely to have been tested included self-pay patients (OR: 0.35, 95% CI: 0.17 to 0.72; ref: those with Medicaid), and those with peritoneal metastases (OR: 0.37, 95% CI: 0.17 to 0.78; ref: those without peritoneal metastases), ovarian metastases (OR: 0.32, 95% CI: 0.11 to 0.98; ref: those without ovarian metastases), or other metastases (OR: 0.47, 95% CI: 0.25 to 0.87; ref: those without other metastases).

Among patients who were *KRAS*-tested, the only factor significantly associated with being tested at diagnosis (Figure 2B) was the calendar time when testing was conducted. Testing at diagnosis was 2.1 times more likely to occur in the first three quarters of 2009 (OR: 2.09, 95% CI: 1.24 to 3.52; ref: those diagnosed in 2008 or earlier) and 6.2 times more likely to occur from the fourth quarter of 2009 onward (OR: 6.15, 95% CI: 2.95 to 12.81; ref: those diagnosed prior to the fourth quarter of 2009) compared to the period before 2009.

## Discussion

Using text-based documents (e.g., physician progress notes) electronically stored within the ACORN Data Warehouse, a text-mining algorithm and a corresponding statistical model was developed to accurately identify patients with mCRC who were and were not tested for *KRAS* genotype. This study is the first to investigate the uptake of *KRAS* testing using EMR data from community-based oncology practices across the US representing all insurance types and multiple health plan types. A standard field to capture whether a patient was *KRAS*-tested and the results of testing is not available in EMR data. In the present study, this was addressed by text mining physician notes and subsequent chart review of those patients identified as *KRAS*-tested. As advances in biomarker identification and testing in cancers of all types continue, oncology-based EMR systems will need to accommodate the capture of this information.

Corresponding to changes in treatment guidelines and US product labels for anti-EGFR agents, *KRAS* testing increased annually between 2008 and 2009 and remained constant through 2011. Nevertheless, despite the increasing frequency of testing appropriate patients for *KRAS* genotype, slightly more than half the patients (52.5%) show no evidence in their medical records of having been *KRAS*-tested. These patients may include those whom physicians consider inappropriate candidates for anti-EGFR therapies, those diagnosed prior to the adoption of *KRAS* testing guidelines, and those with tumors progressing too quickly for *KRAS* genotype-directed treatment decisions. Given that data supporting first-line use of an EGFR inhibitor (cetuximab) over first-line bevacizumab [21] have emerged more recently, most physicians were likely using EGFR inhibitors between 2008 and 2011 in later lines of therapy, and thus, may have waited to test for *KRAS* status (rather than testing at the time of diagnosis). As such, it is possible that patients diagnosed between 2008 and 2011 had not progressed to the point of considering EGFR inhibitor treatment (and accordingly *KRAS*-tested) during the follow-up period included in the dataset. If this is true, then our findings may support a practice of *KRAS* testing at the time of potential EGFR inhibitor use rather than physicians’ lack of knowledge about or uptake of *KRAS* testing in their practice. Indeed, only a fraction (approximately 50%) of patients with mCRC who receive first-line therapy go on to receive second-line therapy and beyond [22,23]. Additionally, the study period for this analysis (2008–2011) occurred before the availability of an FDA-approved *KRAS* diagnostic test in 2012; the availability of such tests may affect the proportion of patients who are *KRAS*-tested in the future.

This analysis suggests that certain patient characteristics may influence whether or not a patient is *KRAS*-tested, but that once a clinician decides to test for *KRAS*, patient characteristics play less of a role in influencing the timing of the decision. In this study population, a substantial proportion of patients were aged 75 years and older; as age increases, patients may be less likely to ever have been *KRAS*-tested. In the model, when the age variable was changed from a quasi-continuous variable to a discrete binary variable (i.e., patients aged 75 and older), the model results were not dramatically different (data not shown).

Although the proportion of tested patients identified as *KRAS*-WT (48%) was slightly lower than previously reported (57%) [18], the 10% of patients with *KRAS*-status unknown (i.e., no evidence of testing) is consistent with what is seen in clinical practice. Unknown status is more likely the result of the pathologist having too little sample to conduct *KRAS* testing rather than the result being unavailable in the chart.

Access to reimbursement (for the costs of both *KRAS* testing and of anti-EGFR therapies) may be a factor affecting *KRAS* testing for appropriate patients, particularly prior to the adoption of the updated treatment guidelines. Additionally, it appears that healthier patients are less likely to be tested than less healthy patients (i.e., those with poor performance status or more comorbidities). One consideration is that patients with poorer performance status and more comorbidities may be better candidates for single-agent EGFR inhibitor therapy than other combination regimens, necessitating *KRAS* testing to determine course of treatment.

Among *KRAS*-tested patients, testing at the time of mCRC diagnosis increased annually from 27.4% in 2008 to 78.4% in 2011, corresponding to current recommendations that patients be *KRAS*-tested at diagnosis of metastatic disease to aid in informing their overall treatment plan. It appears that the community-based oncologists serving this patient population were not only aware of the changes in clinical guidelines (and corresponding changes to EGFR inhibitor product labels), but quickly began implementing *KRAS* testing as part of the workup for most newly diagnosed patients with mCRC.

A limitation of this study is the fact that the contributing oncology practices used one of three EMR systems that feed the Acorn Data Warehouse; this may add variability to which data elements are captured in addition as to how they are captured. An additional data limitation is missing data on care provided outside of the ACORN-contributing practices. The results presented may not be generalizable to community-based oncology practices at large.

## Conclusions

In conclusion, it appears that although there has been an increase in awareness of the need for *KRAS* testing among patients with mCRC (i.e., an increase in the proportion of eligible patients tested; and among those tested, an increase in the proportion of those tested at first diagnosis of metastatic disease), the overall proportion of patients *KRAS*-tested (approximately 50%) could be increased further to improve therapy planning for patients and ultimately improve patient outcomes. Testing rates have likely improved since the period examined within this study (2008–2011) due to further label changes and availability of an FDA-approved test, thus increasing awareness and improving access to testing. Continued education and awareness to promote the importance of *KRAS* testing is warranted, especially as new biomarkers emerge in the treatment of mCRC. As the continuum of care for mCRC evolves and the optimal sequence of available agents is better characterized, maximizing knowledge about predictive and prognostic biomarkers at the time of diagnosis will help clinicians develop short- and long-term individualized therapeutic strategies for their patients.

## Abbreviations

ASCO: American Society for Clinical Oncology
CRC: Colorectal cancer
ECOG: Eastern Cooperative Oncology Group
EGFR: Epidermal growth factor receptor
EMR: Electronic medical record
FDA: Food and Drug Administration
KPS: Karnofsky performance score
mCRC: Metastatic colorectal cancer
NCCN: National Comprehensive Cancer Network
OR: Odds ratio
WT: Wild type
